# The silent defense: Micro-RNA directed defense against HIV-1 replication

**DOI:** 10.1186/1742-4690-4-26

**Published:** 2007-04-12

**Authors:** Ajit Kumar

**Affiliations:** 1 Department of Biochemistry & Molecular Biology, George Washington University, Washington, D.C, USA

## Abstract

MicroRNAs play critical role in regulating gene expression. MicroRNA profile of particular cell type bears the signature of cell type specific gene expression. Given that viral pathogens replicate by evading host defenses, research is now focused on the miRNA-regulated genes that critically regulate HIV-1 propagation in human host cells.

## Background

Ever since the initial report [1] that *C. elegans lin-4 *gene product, a 21 nucleotide non-coding RNA (ncRNA), regulates the expression of *lin-14 *by partial complementarity to several regions within the 3'-UTR of the target *lin-14 *mRNA, RNA-mediated gene silencing (RNAi) has taken on new urgency to understand its role in regulating gene expression in mammalian cells. A recent report in *Science *[2] argues that RNAi limits the replication of HIV-1 in human cells, and that cellular micro-RNAs (miRNAs) contribute to this antiviral response. This report opens the inquiry into exciting new area of virus-host interaction and asks how viral infection overcomes the limitations imposed on virus life cycle by the host miRNA-mediated defenses.

**Figure 1:**
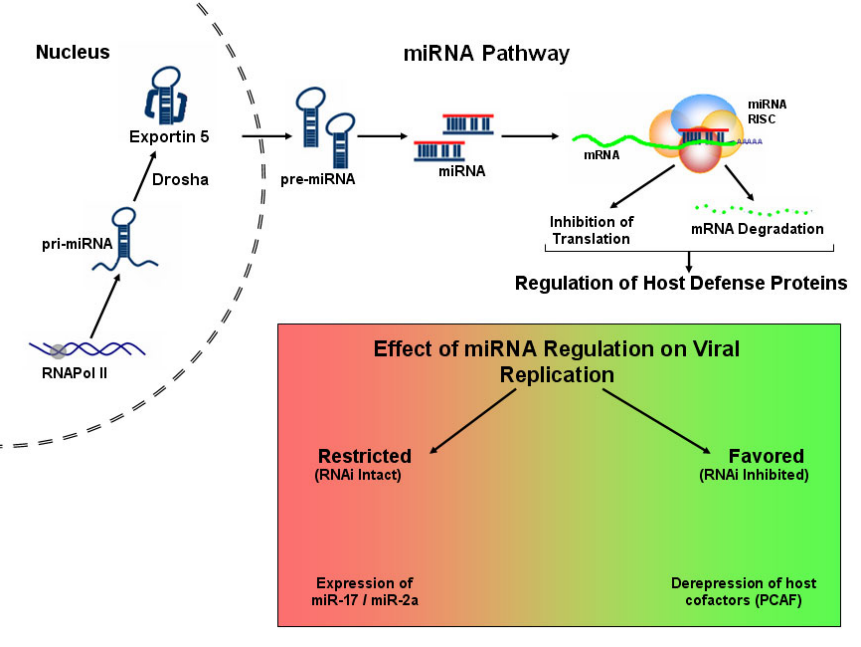
Outlines the restriction on virus replication imposed by host cell RNAi response. Intact RNAi response, or over expression of miR-17/miR-2a severely restricts HIV-1 replication. Host proteins targeted by miRNAs include PCAF, a HIV-1 Tat-cofactor, its expression favors HIV-1 replication.

Nearly 500 human genes are known to encode ~21 nucleotide miRNAs, which are initially transcribed by RNA Polymerase II as primary (pri-miRNA) that are processed in the nucleus by RNase type III Drosha into precursor (pre-miRNA) and exported to the cytoplasm by exportin 5, to be secondarily processed into miRNA duplexes by the cytoplasmic RNAse type III Dicer. The resulting miRNA duplexes are incorporated into the RNA-Induced Silencing Complex (RISC) where one of the miRNA strands, the 'passenger' is degraded, while the 'guide' miRNA is guided to the target mRNA to either degrade (in case of perfect base complementarity) or to block translation (in case of imperfect sequence complementarity between the miRNA 'seed' sequence and the target mRNA). This general version of miRNA action (Figure 1) may not be universally true in all cases; nevertheless, examining the miRNA-targeted genes has allowed a detailed understanding of the host response to the stress induced by viral infection.

Triboulet et al., [2] show that reducing the Drosha or Dicer levels in host cells allowed faster kinetics of HIV-1 production. One could quibble with the fact that siRNA-mediated knock down of Drosha and Dicer levels in the host cells may be considered a 'blunt tool'. The results nevertheless argue that the intact RNAi pathway of the host keeps virus replication in check. The question is how? What are the miRNA mediators of host defense that HIV needs to overcome in order to propagate? The authors analyzed miRNA landscape in uninfected and HIV-1 infected cells and found that several miRNAs (miR-122, miR-370, miR-373 and miR-297) are up regulated during HIV replication. The authors noted that these up regulated miRNAs are not normally expressed in T-cells. Could these up regulated miRNAs modulate expression of host genes related to basal response to virus replication? This report does not pursue the role of host genes that are targeted by the miRNAs up-regulated during HIV-1 replication.

The experiments discussed by Triboulet et al, however do emphasize the importance of the miR-17/92 cluster that is down-regulated during HIV replication. The down-regulated miRNAs include, miR-17-5p/3p, miR-18, miR-19a, miR-2a, miR196-1 and miR-92-1. Significantly, host proteins targeted by the miR-17/92 cluster include histone acetylase, PCAF; PCAF has been shown to be an important co-factor in Tat-transactivation and HIV-1 replication. There are four potential targets within the PCAF 3'-UTR for miR-17-5p and miR-2a binding which could lead to translational inhibition of the PCAF-transcript. Over expression of miR-17-5p or miR-2a resulted in dramatic reduction of HIV-1 production. Importantly, the restoration of PCAF protein levels, as indicated by the expression of PCAF cDNA vectors lacking the 3'-UTR, was sufficient to relieve the suppression of HIV-1 production imposed by the miRNAs. One could argue that histone acetylation is a general positive regulator of transcription; a point supported by the observation that the repressive effect of RNAi on HIV-1 replication was also seen in latently infected U1 cells which express a mutant Tat and are unable to efficiently activate HIV-1 LTR [3].

miRNAs expressed in a particular cell type bear a signature of specific gene expression pattern of that cell type [4]. The repertoire of expressed miRNAs varies from one cell type to another. Although the basic steps in miRNA biogenesis are known, it is less clear how miRNA expression is regulated in different cell types. Importantly, it is largely unknown how virus replication influences the abundance and the distribution of miRNAs within the host cell. Given the importance of miRNAs as critical effectors that modulate specific protein levels, changes in miRNA landscape during virus replication is a promising approach to understand molecular regulation of host defenses and the attempt by viruses to overcome host defense during infection.

The range of interactions possible through miRNA-mRNA cross-talk during host-virus interaction is complex [5]. Successful viruses effectively use the host machinery to express viral proteins; while effective hosts limit viral propagation by mobilizing innate and adaptive antiviral defenses. miRNAs clearly have a central role in modulating gene expression during pathogen-host interaction. There have been reports that predict candidate miRNAs of viral origin (vmiRNAs) that would target host genes to facilitate virus replication [6]. As well, there are predicted target sites for human encoded miRNAs in HIV genes [7]. In a recent report Konstantinova et al. [8] constructed HIV-1 which expresses a stable 300 bp long hairpin RNA (lhRNA) targeted to Nef and LTR sequences and found that this viral construction induce antiviral effects against wild-type HIV-1 in *trans*, perhaps through a sequence-specific RNAi mechanism, although direct data supporting that were not demonstrated. This finding is consistent with the notion that mammalian cells are fully competent for processing of miRNA, siRNA, or shRNA sequences within the context of an HIV-1 genome.

Rapid progress in miRNA research is currently hampered by lack of accuracy in predictions of the physiologically relevant transcripts targeted by miRNAs. Indeed, although computer based prediction programs are easily accessible, empirical results suggest that many in silico predictions of miRNA targeted genes will have to be experimentally validated in biological assays. The complexity of the system is in part due to the finding that one miRNA can have binding sites in multiple targets and one transcript can be attacked by many discrete miRNAs [9]. Computational algorithms for miRNA prediction that rely heavily on sequence conservation may prove to be inadequate for viruses. A more useful strategy may incorporate embedded secondary signals in either the RNA itself, or the structure of the resulting RNA-RNA or RNA-protein in the RISC complex to carry out the analogue action required for accurate miRNA targeting [10]. Examples are complexes of RNA modifying enzymes which act at a site adjacent to and determined by the position of the snoRNA:target interaction [11] and the RISC complexes [12].

Viral miRNAs, unlike their vertebrate counterparts, do not share a high level of homology, even within members of the same family or with that of the host. RNA viruses as compared to the DNA viruses, since their RNA genome is more susceptible to attack by RNAi, are less likely to maintain RNAi-targeted sites. There is however an interesting example [13] of accumulation of HCV RNA induced by liver specific miR-122. This novel mechanism involving the interaction of miR-122 and the 5'UTR of HCV RNA may have evolved in parallel with the highly conserved 5'UTR secondary structure of HCV RNA essential for translational control of viral proteins. In another example [14], mammalian microRNA, miR-32 has been shown to restrict the accumulation of the retrovirus, primate foamy virus type-1 (PFV-1, akin to human HIV). Cellular miRNA, miR-32 efficiently inhibits the replication of PFV-1 by hybridizing with the 3'UTR of viral mRNAs [15]. Remarkably, HIV-1 Tat has been shown to inhibit Dicer activity, independently of its transcriptional function [16,17]. Studies on the involvement of miRNAs in regulation of innate immune response showed that miR-146a/b may function as novel negative regulators that fine-tune the immune response [18]. Furthermore, post-transcriptional repression of gene expression mediated by miRNA appears to be subject to regulation by physiological stress in human cells [19].

These are exciting times for non-coding RNAs (ncRNAs) that come not only in small forms. In the coming period, one can expect to gain novel insights into the regulation of mammalian gene expression by a better reading of the language of ncRNAs.
